# Genome-Wide Linkage Analysis of Cardiovascular Disease Biomarkers in a Large, Multigenerational Family

**DOI:** 10.1371/journal.pone.0071779

**Published:** 2013-08-02

**Authors:** Daniel Nolan, William E. Kraus, Elizabeth Hauser, Yi-Ju Li, Dana K. Thompson, Jessica Johnson, Hsiang-Cheng Chen, Sarah Nelson, Carol Haynes, Simon G. Gregory, Virginia B. Kraus, Svati H. Shah

**Affiliations:** 1 Center for Human Genetics, Duke University, Durham, North Carolina, United States of America; 2 Department of Medicine, Duke University, Durham, North Carolina, United States of America; 3 Division of Rheumatology, Immunology and Allergy, Tri-Service General Hospital, Taipei, Taiwan; 4 Department of Biostatistics, University of Washington, Seattle, Washington, United States of America; University of California, Riverside, United States of America

## Abstract

Given the importance of cardiovascular disease (CVD) to public health and the demonstrated heritability of both disease status and its related risk factors, identifying the genetic variation underlying these susceptibilities is a critical step in understanding the pathogenesis of CVD and informing prevention and treatment strategies. Although one can look for genetic variation underlying susceptibility to CVD *per se*, it can be difficult to define the disease phenotype for such a qualitative analysis and CVD itself represents a convergence of diverse etiologic pathways. Alternatively, one can study the genetics of intermediate traits that are known risk factors for CVD, which can be measured quantitatively. Using the latter strategy, we have measured 21 cardiovascular-related biomarkers in an extended multigenerational pedigree, the CARRIAGE family (Carolinas Region Interaction of Aging, Genes, and Environment). These biomarkers belong to inflammatory and immune, connective tissue, lipid, and hemostasis pathways. Of these, 18 met our quality control standards. Using the pedigree and biomarker data, we have estimated the broad sense heritability (H2) of each biomarker (ranging from 0.09–0.56). A genome-wide panel of 6,015 SNPs was used subsequently to map these biomarkers as quantitative traits. Four showed noteworthy evidence for linkage in multipoint analysis (LOD score ≥ 2.6): paraoxonase (chromosome 8p11, 21), the chemokine RANTES (22q13.33), matrix metalloproteinase 3 (MMP3, 17p13.3), and granulocyte colony stimulating factor (GCSF, 8q22.1). Identifying the causal variation underlying each linkage score will help to unravel the genetic architecture of these quantitative traits and, by extension, the genetic architecture of cardiovascular risk.

## Introduction

Cardiovascular disease (CVD) is the leading cause of death, accounting for over 500,000 deaths per year in the United States and greater than seven million deaths worldwide. It has been estimated that over 82 million Americans are afflicted with at least one form of CVD and over 16 million Americans are afflicted with clinically significant CVD [1]. There are many well accepted CVD risk factors including common metabolic conditions (hypertension, dyslipidemia, metabolic syndrome, diabetes), behavioral factors (smoking, sedentary lifestyle), and non-modifiable factors (sex, age). Thus CVD is etiologically complex with independent genetic influences on CVD susceptibility as well as genetic influences on CVD-related risk factors. 

Given the importance of CVD to public health and the demonstrated heritability of both disease status and its related clinical risk factors, identifying the genetic variation underlying these susceptibilities is a critical step in understanding the pathogenesis of CVD to inform prevention and treatment strategies. Although many studies have examined genetic variation underlying susceptibility to CVD, it can be difficult to define the disease phenotype for such a qualitative analysis because CVD likely represents an end convergence of diverse biological etiologic pathways, reflects genetic factors operating through these different pathways which may be different than factors influencing CVD directly, and results from an incompletely understood interaction of genetic and environmental factors. Alternatively, one can study the genetics of CVD through a quantitation approach that uses intermediate traits that are known risk factors for CVD and that can be measured quantitatively; we have used this method successfully for identification of osteoarthritis genes [2].

As quantitative traits we measured 21 biomarkers with relevance to CVD. We present herein the subsequent quantitative trait loci (QTL) mapping of these disease-related intermediate traits in an extended multigenerational pedigree as part of the CARRIAGE (Carolinas Region Interaction of Aging, Genes, and Environment) study. The CARRIAGE family is one of the most extensively pedigreed families in the U.S., comprising 10 generations and 3,327 individuals descended from a single founding couple born in the 18^th^ century, and composed of primarily African and Native American ethnic origin. The potential for reduced genetic heterogeneity within extended pedigrees, such as this one, facilitates linkage analysis [3]. This family has a reduced prevalence of CVD relative to the general population with 15% of studied family members having at least one cardiovascular condition compared to approximately 33% in the US population [1]. Thus the genetic effects identified for the CVD-risk biomarkers reflect effects in a sample not ascertained for CVD conditions.

## Materials and Methods

### Study population: the CARRIAGE family

The CARRIAGE family data collection has been described previously [2]. For this study, we have used a detailed ascertainment of 350 family members from whom blood samples were available, as previously reported [2]. Written informed consent was obtained from each participant, and the study was approved by the Duke Institutional Review Board. All information and work was conducted under a Federal Certificate of Confidentiality to ensure the privacy of each participating member’s clinical and genetic data.

### Selection and measurement of serum biomarkers

The biomarkers selected belong to important known pathways involved in CVD risk: (1) inflammatory and immune (C reactive protein [hsCRP], monocyte chemotactic protein one [MCP1], Regulated upon Activation, Normal T-cell Expressed, and Secreted [RANTES], granulocyte colony stimulating factor [GCSF], interleukin eight [IL-8], TNF-related apoptosis-inducing ligand [TRAIL], interleukin-six [IL-6], interleukin-two [IL-2], interleukin-one beta [IL-1β], interleukin-one receptor antagonist [IL1RA], tumor necrosis factor receptor two [TNFR2], tumor necrosis factor receptor one [TNFR1], and tumor necrosis factor alpha [TNFα]); (2) connective tissue (vascular endothelial growth factor [VEGF], matrix metalloproteinase three [MMP3], and brain derived neurotrophic factor [BDNF]); (3) lipid (paraoxonase, adiponectin, and leptin); (4) hemostasis (D-dimer); and (5) metabolic pathways (glycated albumin [GSP]).

The procedures for biomarker quantification are described below (unless otherwise stated, all kits were used to analyze serum according to the manufacturer’s instructions). Total adiponectin was measured by enzyme-linked immunosorbent assay (ELISA) using a kit from ALPCO Diagnostics (Salem, NH), with samples diluted 1:1000. D-dimer was measured by ELISA using a kit from American Diagnostics (Stamford, CT), with plasma samples diluted 1:50. IL-6 was measured using a high sensitivity immunoassay from MesoScale Discovery (Gaithersburg, Maryland). Leptin was measured by ELISA using a kit from Millipore Corporation (Billerica, MA), with 25µl of sample and standards used. MMP-3 was measured by ELISA using a kit from Invitrogen Corporation. Paraoxonase activity was measured (nmol product formed/min/ml) via a kit from Invitrogen Corporation; serum samples were diluted 1:100 and the reaction was stopped after 60 minutes and fluorescence measured with an excitation wavelength of 360nM and an emission wavelength of 465nM. TRAIL was measured by ELISA using a kit from Invitrogen. High sensitivity CRP was detected by solid-phase ELISA (MAGIWEL; UBI, Mountain View, CA). GSP was detected via the specific enzymatic method using reagents from DIAZYME (Poway, CA). For individual assays, samples were measured in duplicate. The inter- and intra-assay percent coefficients of variation for the individual assays are as follows: adiponectin (2.31, 8.84), D-dimer (4.32, 9.35), IL-6 (4.36, 16.9), leptin (5.61, 6.46), MMP-3 (2.59, 11.04), paraoxonase (2.58. 15.9), and TRAIL (3.74, 18.0). A Luminex Panel from Invitrogen Corporation (Carlsbad, CA) was used to measure BDNF, GCSF, IL-1RA, IL-1β, IL-2, IL-8, MCP1, RANTES, TNFα, TNFR1, TNFR2, and VEGF. For Luminex, samples were assayed in individual wells, from which at least one hundred beads were analyzed. Biomarkers with greater than 25% of the measured sample concentrations at or below the limit of quantification (LOQ) were not further analyzed (BDNP, IL2, and IL1β). For the remaining biomarkers, samples with concentrations below LOQ (representing ≤2% of samples for any given biomarker, Table S1) were assigned a value of ½ LOQ.

### Genome-wide genotyping

DNA was isolated and quantified according to standard protocols, as previously described [2]. Genome-wide genotyping was performed using the Infinium HumanLinkage-12 Genotyping BeadChip (Illumina, San Diego, CA). This assay includes 6,090 single-nucleotide polymorphism (SNP) markers with an average marker density of one per 0.58 centiMorgans (cM). The quality control measures included: genotyping two control samples from the Centre d’Étude du Polymorphisme Humain (CEPH) and requirement of a genotype call-rate ≥ 98% for each SNP. A total of 6,015 of the 6,090 SNPs (98.8%) met this quality control standard. Two individuals were excluded from the analysis due to overall low call rates (<95%). In addition, Mendelian inconsistencies, Hardy-Weinberg equilibrium and errors in sex assignment were examined. Specifically, the genetic analysis package RELPAIR [4] was used to verify the reported family relationships. Twenty-four unrelated individuals were excluded from any further analysis. One individual was removed due to gender error using PLINK [5]. The genetic analysis package VITESSE [6] was used to identify Mendelian errors in genotyping across generations. Of the 2+ million genotypes across all SNPs typed on these individuals, there were a total of 188 Mendelian errors. These genotypes were excluded from further analysis. SNPs out of deviation with Hardy-Weinberg equilibrium (p<0.0001) were removed and not further analyzed. The deCODE genetic map was used to position the markers (deCODE Genetics, Reykjavik, Iceland).

### Statistical analysis

#### Heritability

The broad-sense heritability (H2) of each biomarker was estimated using the Sequential Oligogenic Linkage Analysis Routines (SOLAR) [7] with an adjustment for age and sex. Broad-sense heritability includes the aggregate genetic variance resulting from additive, epistatic, dominant, maternal, and paternal genetic effects. As the assumption of normality is very important in variance components analysis several transformations were used to achieve approximate normality [8]. All biomarker levels were log transformed to approximate a normal distribution. After log transformation, there was residual kurtosis for nine of the biomarkers (D-dimer, GSP, IL1RA, IL-6, RANTES, TNFα, TNFR1, TNFR2, and TRAIL). Extreme outliers were sequentially removed for these biomarkers, starting with the removal of any values greater than or equal to four standard deviations from the mean. If the marker distribution still had significant kurtosis, then any values greater than or equal to three standard deviations from the mean were removed. This process resulted in residual kurtosis remaining for two markers (TNFα and IL-6) and these markers were analyzed using the “lodadj” command implemented in SOLAR to compensate for this lack of normality. Three traits (adiponectin, TNFα, and TNFR1) required rescaling to approximate normality and in those cases the trait value was multiplied by a factor ranging from 2.3–5.8. A polygenic variance components model was fitted and used as the foundation for subsequent linkage analysis.

#### Quantitative trait linkage analysis

Two-point and multipoint genome-wide linkage scans were performed for all autosomes using 18 CVD-related biomarkers as quantitative traits. As recommended for multigenerational pedigrees, linkage between each of the biomarker traits and marker loci was tested by maximum-likelihood methods using a variance components model [9]. The size and complexity of the CARRIAGE pedigree necessitated first computing the identity-by-descent (IBD) probabilities for each pair of individuals at each marker using the Markov-Chain Monte-Carlo (MCMC) algorithm implemented in the Loki analysis package [10]. Linkage was interpreted as significant if the logarithm of odds (LOD) score was ≥ 3.0, “noteworthy” if the LOD score was ≥ 2.6, and scores ≥ 2.0 were identified as “interesting”.

## Results

**Figure 1:**
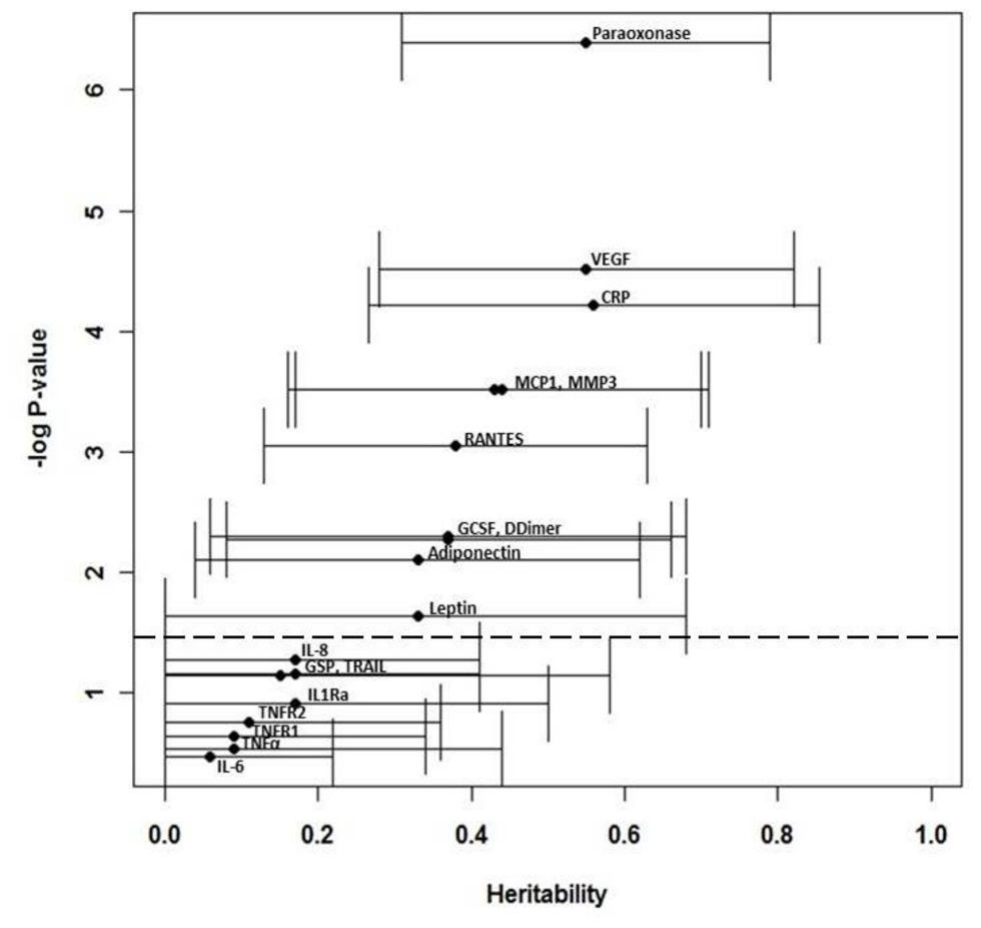
Heritabilities of measured CVD biomarkers. Presented is the distribution of heritability and its corresponding p-value for all 18 biomarkers, with the heritability estimate on the X-axis and the –log base 10 of the associated p-value on the Y-axis. The 95% confidence interval is represented by a horizontal error bar. The threshold for significance is represented by a dashed line.

Baseline clinical characteristics of the study population are presented in Table 1. Mean levels for each of the 18 biomarkers are listed in Table 2, and are similar to those seen in the general American population, consistent with the fact that this population was not ascertained for any particular disease or metabolic phenotype. Eleven of the 18 biomarkers showed nominally statistically significant heritability (p<0.05, Figure 1), with heritability estimates ranging from 0.33 (leptin, p=0.02, SE=0.18) to 0.56 (hsCRP, p=0.00006, SE=0.15).

**Table 1:** Baseline clinical characteristics of the CARRIAGE cohort.

Clinical characteristic	Mean (SD)*	Percent*
Sex (% female)		66%
Hypertension		42%
Diabetes		15%
Smoker		78%
Body mass index	31.1 (6.8)	
Age, years	54.1 (15.3)	
Low density lipoprotein (LDL) cholesterol, mg/dL	112.2 (36.5)	
High density lipoprotein (HDL) cholesterol, mg/dL	48.0 (14.1)	
Triglycerides, mg/dL	137.2 (90.9)	

* Quantitative traits presented as mean (standard deviation); discrete traits presented as percent prevalence.

**Table 2:** Summary statistics for CVD biomarkers.

Biomarker	Units	Mean (SD)	Max	Min
Adiponectin	ng/mL	13044.2 (6404.3)	52794.0	4462.7
hsCRP	ng/mL	8.1 (1.4)	11.4	3.8
DDIMER	ng/mL	703.7 (1015.0)	9057.0	78.3
GCSF	pg/mL	314.6 (388.5)	3225.0	20.6
GSP	µmol/L	227.3 (58.1)	675.1	106.0
IL1RA	pg/mL	4502.1 (10758.7)	110666.0	158.0
IL6	pg/mL	1.5 (5.4)	80	.03
IL8	pg/mL	85.1 (50.8)	542.6	12.2
Leptin	ng/mL	30.4 (26.2)	304.1	0.3
MCP1	pg/mL	2601.5 (1601.2)	11840.6	151.7
MMP3	ng/mL	7.2 (7.3)	79.1	1.0
Paraoxonase	nmol/min/L	11.6 (4.1)	27.4	2.3
RANTES	pg/mL	9584.7 (17167.6)	243369.0	819.7
TNFα	pg/mL	29.5 (88.8)	1240.7	5.9
TNFR1	pg/mL	5799.2 (3444.5)	25533.2	533.0
TNFR2	pg/mL	2485.7 (1482.1)	13936.6	136.0
TRAIL	pg/mL	491.2 (195.2)	2012.9	86.9
VEGF	pg/mL	227.6 (233.2)	1685.6	32.9

The unit of measurement, mean, standard deviation, maximum, and minimum values for each biomarker are given.

Two point linkage analysis revealed that the highest LOD score was for VEGF (chromosome 4p14, SNP rs790142, LOD=2.6). In addition, eight additional SNPs were identified as interesting based on evidence of linkage with LOD ≥ 2.0 for five biomarkers (VEGF, hsCRP, MCP-1, D-dimer, and paraoxonase, Table 3). The maximum two point LOD score obtained for each biomarker per chromosome is presented in Table S2.

**Table 3:** Results for genome-wide linkage, two-point LOD scores.

Biomarker	LOD	SNP	Chromosome	Gene*
hsCRP	2.10	RS1419607	7	Intergenic (*POT1, GRM8*)
	2.02	RS728919	11	*NAA40*
	2.49	RS7240966	18	Intergenic (*CTIF*)
D-dimer	2.18	RS2780701	9	*SYK*
MCP1	2.14	RS857819	1	Intergenic (*OR6N1*)
Paraoxonase	2.10	RS491603	1	Intergenic (*EIF2C3*)
	2.00	RS726455	13	Intergenic (*SOX1*)
	2.25	RS234	7	Intergenic (*CDHR3*)
VEGF	2.56	RS790142	4	Intergenic (*NSUN7*)

Results for genomic regions with noteworthy (LOD ≥ 2.6) or interesting (LOD ≥ 2.0) two-point LOD scores are presented. The biomarker name is given, followed by the two-point LOD score, SNP rs number, chromosome and gene name.

*if SNP is intergenic, the closest gene(s) is listed in parentheses.

**Figure 2:**
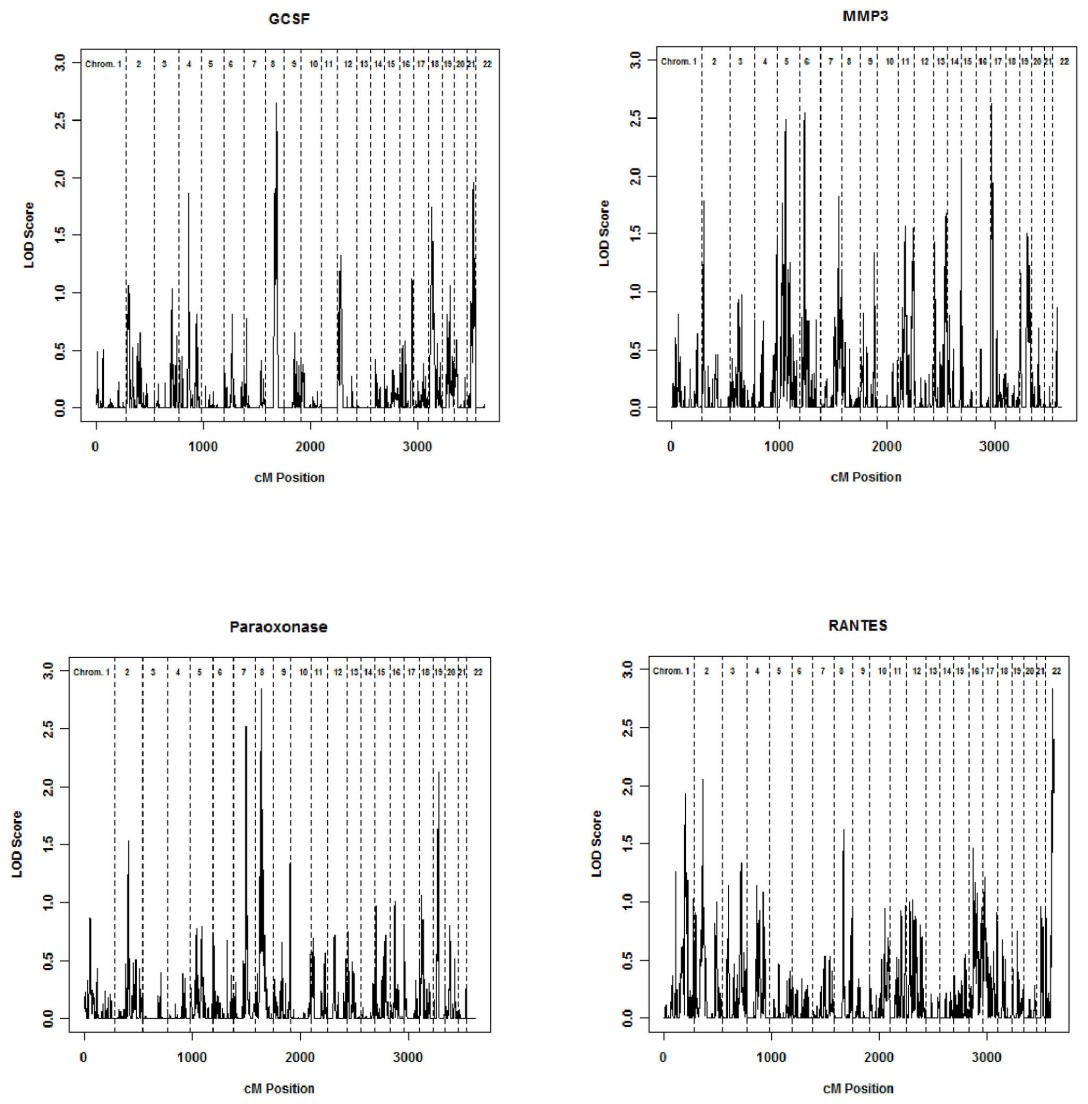
Multipoint genome-wide linkage scans for GCSF, MMP3, paraoxonase, and RANTES. Vertical dashed lines represent boundaries between chromosomal regions and the cumulative cM position is indicated on the X axis.

In multipoint analyses, we identified noteworthy (LOD ≥ 2.6) QTL for four of the 18 CVD biomarkers: paraoxonase, RANTES, MMP3, and GCSF (Figure 2a–d, Table 4, Figure S1a–d, Figure S2a–n). Specifically, paraoxonase had noteworthy or interesting evidence for linkage at three locations: 8p11.21 (multipoint LOD [MLOD] 2.8), 7q22.1 (MLOD 2.5), and 19q12 (MLOD 2.1). RANTES had evidence for linkage at one location, 22q13.33 (MLOD 2.8). MMP3 had evidence for linkage at three locations: 17p13.3 (MLOD 2.6), 6p22.3 (MLOD 2.5), and 5q12.3 (MLOD 2.5). Finally, GCSF had evidence for linkage at one location, 8q22.1 (MLOD 2.6). Additionally, six of the remaining 14 biomarkers had interesting results based on evidence for linkage with a LOD ≥ 2.0 (Table 4).

**Table 4:** Chromosomal locations for highest multipoint LOD scores.

Biomarker	Region	SNPs*	Region	SNPs*
	LOD 2.6-3.0		LOD 2.0-2.5	
Paraoxonase	8p11.21(2.8)	RS8685, RS749540	7q22.1(2.5) 19q12(2.1)	RS1229540, RS234 RS7250192, RS2194198
RANTES	22q13.33(2.8)	RS7410750, RS10451		
MMP3	17p13.3(2.6)	RS216219, RS12746	6p22.3(2.5) 5q12.3(2.5)	RS965037, RS1264451 RS1020661, RS164561
GCSF	8q22.1(2.6)	RS1051624, RS951826		
GSP			13q13.2(2.4)	RS306395, RS668103
TRAIL			1q44(2.3)	RS164561, RS2027432
hsCRP			7q32.3(2.2)	RS12217, RS1371463
MCP1			9q34.3(2.1) 1q22(2.0)	RS3132332, RS7357733 RS13320, RS10918078
D-dimer			9q34.2(2.1)	RS10818768, RS7860423
Adiponectin			11p15.5(2.1)	RS741737, RS879114

Presented is a summary of all multipoint LOD score results greater than 2.0 obtained in the genome scan (multipoint LOD score in parentheses), with the SNPs flanking the 1 LOD down interval.

* SNPs flanking 1 LOD down interval around peak marker in QTL.

## Discussion

Using a large, multiethnic, multigenerational extended family, we have successfully identified QTL using CVD-related inflammatory and metabolic biomarkers. These QTL may harbor genes for genetic susceptibility for CVD mediated through these biological pathways. Most of the biomarkers were also found to be significantly heritable (p<0.05); we believe this to be a novel finding for GCSF and MMP3, while the other heritable biomarkers have support from the previous literature [11–13]. Our unique study design, employing a single extended multigenerational family not ascertained on CVD and thus with a burden of CVD and related risk factors similar to or less than the United States population, facilitates extension of these findings to the general population with a common burden of risk factors.

The strongest QTL was for paraoxonase, an enzyme associated with high density lipoprotein (HDL) cholesterol that inhibits oxidation of low density lipoprotein (LDL) cholesterol. Oxidized LDL is important in the atherosclerotic process [14], and it has been shown that low paraoxonase levels are associated with increased risk of myocardial infarction [15]. In our study, there were several interesting QTL for paraoxonase levels, the strongest of which was on chromosome 8p11, 21, a genomic region which contains genes encoding several transcription factors (*ZMAT4*, *NKX6-3*, *IKBKB*, *THAP1*, and *RNF170*) and an enzyme involved in post-translational modification (*FNTA*); all of these could be plausible candidates for regulators *in trans* of paraoxonase levels. Previously published genome-wide linkage studies have reported linkage to other parts of the genome, including the physical locus for the paraoxonase gene cluster (*PON1, PON2, PON3*), on chromosome 7q, but those studies did not report evidence at any of the other loci detected in our study [16]. The linkage peak we identified at 7q22.1 occurs at the paraoxonase gene cluster and serves as a proof of principle for the accuracy of our analyses, even in this complex family-based study. The paraoxonase gene cluster regulates paraoxonase levels and *PON1* and *PON3* genetic variants are associated with CVD risk [17,18], supporting the concept of using intermediate disease-related markers as quantitative genetic traits for disease gene mapping. The regions linked to paraoxonase levels are also linked to other disease traits. For example, the 7q22 region, that contains the genes reelin (*RELN*) and leptin (*LEP*), is linked to several conditions including: osteoarthritis [19], autism [20], body mass index [21,22], and dilated cardiomyopathy [23]. The region on 19q12, that contains the gene for the ryanodine receptor [*RYR1*] [24] (a class of intracellular calcium channels found primarily in cardiac muscle), are linked to paraoxonase levels as well as linked to waist circumference [25], BMI [26], resistance to muscle fatigue [27], essential hypertension [28], prostate cancer [29], maturity onset diabetes of the young (MODY) [30], and malignant hyperthermia [31].

The chemotactic cytokine RANTES recruits T-cells, eosinophils, and basophils to sites of inflammation and is therefore a likely participant in CVD through the contribution of inflammation to atherosclerotic plaque formation and response to plaque rupture. The region around the LOD score peak for RANTES (22q13) contains the gene *IL17REL* (IL17 Receptor E Like), which was recently identified in a genome-wide association study (GWAS) for ulcerative colitis [32]. The ligands for this receptor are unknown but, due to its high sequence homology to *IL17RE*, it is very likely that it binds a cytokine and has an immunologic role and thus could affect levels of RANTES. This locus is linked to several traits including pulse pressure [33], bone mineral density [34], serum creatinine [35], rheumatoid arthritis [36], schizophrenia [37], height [38], and breast cancer [39].

Matrix metalloproteinase (MMP3) plasma concentrations are linked to increased risk of plaque rupture and, thus, to myocardial infarction [40]. In our study, the strongest evidence for linkage to MMP3 levels was found within a relatively gene-dense region of chromosome 17, which contains several interesting candidate genes including a scavenger receptor for LDL (*SCARF1*) and a highly conserved intracellular signaling protein (*YWHAE*). In addition, this region is linked to ventricular hypertrophy [41], childhood obesity [42], and rheumatoid arthritis [43], among others. The chromosome 6p22 region we found linked to MMP3 levels is linked to sarcoidosis [44], schizophrenia [45], reading ability [45], pulse pressure [46], and early onset myocardial infarction [47]. The third peak for MMP3 in the 5q12 region is linked to low density lipoprotein size [48] and stroke [49].

GCSF is a growth factor and a cytokine which, in addition to its obvious role in promoting the growth of granulocytes, also promotes the growth of stem cells and their release from the bone marrow and has been implicated in the response of vascular endothelial cells to oxidative stress [50]. We observed the strongest evidence for linkage to GCSF levels at chromosome 8q22, which contains the hematopoietic transcription factor *RUNX1T1*. Although there are no data specifically suggesting this, one might postulate regulatory networks in hematopoiesis wherein alterations in *RUNX1T1* function or expression impact GCSF levels. Other studies have reported linkage at 8q22 for hypertension [51], dihydrotestosterone levels [52], and Tourette’s syndrome [53], although the actual genes have not yet been identified.

The significant heritabilities of these CVD biomarkers are not necessarily surprising, as they are potential predictors or risk factors of CVD, and CVD itself has a relatively strong genetic component (i.e. heritability of 0.38-0.57 [54]). A significant heritability means genetic components can explain part of the variation in the trait. Such components can, in theory, be mapped; however, it does not necessarily mean that the underlying genetic model will allow those components to be easily detected via current techniques. For example, as is the case for human height, a trait may have a very high heritability that is due to the additive behavior of many genes, each of which contributes a small amount to the trait variability; such a trait would be very difficult to map with current QTL methods. Thus, it is interesting to note that of the 11 biomarkers with significant heritability point estimates, eight showed interesting results with evidence for linkage to one or more genomic regions, (LOD ≥ 2.0). This suggests that the genetic architecture governing levels of these biomarkers may be amenable to mapping and potentially eventual positional cloning. Interestingly, the biomarker TRAIL did not have significant heritability estimates and yet had some interesting results for evidence for linkage. This discrepancy is likely to result from a higher intra-assay coefficient of variation which may impact the estimate of the heritability as well as reduce power of the linkage analysis.

We can make some specific inferences about this genetic architecture by examining the position of linkage peaks relative to those loci that directly encode a biomarker. For example, the enzyme paraoxonase is encoded by a cluster of genes (PON1-3) on chromosome 7q. In our study, we had linkage to this region on 7q, suggesting regulation *in cis* and possibly allelic variation in the *PON* genes themselves, as has been previously observed [18]. However, the strongest LOD score related to paraoxonase in our study was not linked to the paraoxonase locus on 7q but was found on 8p11. This suggests that regulation *in trans* could contribute in some significant way to variability in paraoxonase levels. The role of regulation *in trans* is underscored by the fact that, of the remaining LOD scores ≥ 2.6 (MMP3, RANTES, and GCSF), none were coincident with the physical loci encoding the biomarker. Thus, just by examining the locations of our LOD scores relative to the loci directly encoding the biomarker in question, we were able to unravel some of the genetic architecture of the trait. Interestingly, we did not find overlap between the only interesting QTL for hsCRP in our study (chromosome 7q32, LOD 2.2) with other published genomic regions linked to and/or associated with hsCRP levels (1p22 [55], 1q23 [55], 2q14 [55], 10q21 [55], 11p11-p13 [56], 11q14 [55], 12p11 [57], 12q15 [57], 19q12 [55], and 20q13 [58]); CRP levels (1p22 [55], 1q23 [55], 2q14 [55], 10q21 [55], 11p11-p13 [56], 11q14 [55], 12p11 [57], 12q15 [57], 19q12 [55], and 20q13 [58]); this may be due to the fact that our population was not ascertained based on disease status, locus heterogeneity, and/or the nonspecific nature of hsCRP as a biomarker of inflammation.

There are limitations to the current approach. Namely, the study was conducted in a population with a specific ancestry, primarily African and Native American and therefore the genetic loci detected in our genome screen may not be applicable to other ethnicities and populations. However, in some ways this ‘limitation’ is also a great strength, as African Americans are an understudied population at high risk for CVD. Furthermore, it is not known to what extent a primarily African American sample would be expected to share causal variation underlying biomarker levels with another ethnic group. In addition, it is possible that the presence of linkage disequilibrium (LD) between SNPs can inflate LOD scores and, as our population is of mixed African American descent, there exists the potential for the significant LD inherent in recently admixed populations. However, the SNPs selected for the genome scan were designed to limit LD between markers and there was no significant LD (r^2^ > 0.4) between any of the SNPs in our most significant linkage peaks (data not shown). The genome scans themselves were conducted under the assumption that the biomarkers were not correlated and that each genome scan was a set of independent tests. However, the level of inter-marker correlations between biomarkers could invalidate that assumption and imposes a multiple testing burden that could influence the significance interpretation of our LOD scores. Identifying all MLODs greater than or equal to two as “interesting” resulted in 15 loci, translating to only 3.7% of the MLODS across all biomarkers and the 22 autosomes. Even so, any of the 15 highlighted results could be type I errors. However, our intent was to highlight the regions providing the most evidence for linkage in our study. Finally, there is no single method that allows for maximal power while using all measurements of the quantitative trait, including extreme outliers. In the current study, we have elected to remove extreme values at either range of the quantitative measure, thus creating distributions that are closer to normality. In so doing, it is possible that some biologically meaningful information has been lost. Other analytic methods (such as the lodadj option in SOLAR) would allow those values to be included, but could also introduce compromises in power, particularly when the overall data does correspond well to a normal distribution.

The results presented here can advance our understanding of CVD in several ways depending on how the biomarker in question relates to the pathogenesis of CVD. First, if the biomarker is a biochemical mediator of CVD, i.e. the biomarker is causal in CVD pathogenesis, then identifying the genes that modify levels of this biomarker could identify targets for development of therapeutic targeting of those genes as well as serving as CVD biomarkers themselves. Second, if the biomarkers are a result of CVD, then the genetic variants responsible for their levels should be genetic risk factors for disease severity. In that case, identifying those variants will advance efforts to predict CVD burden or risk of progression by genotyping. Each of the four biomarkers with LOD scores ≥ 2.6 has been implicated in some way with the pathogenesis or risk of CVD. Fine-mapping of these QTL to identify the responsible gene(s), subsequent evaluation of those genetic markers for association with CVD, and validation in further cohorts are necessary. The identification of the putative causal variants underlying the linkage results for these four biomarkers will not only advance our understanding of cardiovascular risk but hopefully serve as a model for the study of other complex diseases via the genetic dissection of intermediate traits.

## Supporting Information

Table S1 **Percent of samples measured as below lower limits of quantification for a given biomarker assay.**
(DOCX)

Table S2 **Displayed are the maximum two-point LOD score at theta equal zero (MaxLOD) for each biomarker by chromosome, with the name of the probe (RS number), the centimorgan (cM) position of the probe, and the gene annotation for the SNP (or the closest gene for intergenic SNPs).**
(DOCX)

Figure S1 **Chromosome linkage plots for the most significant multipoint linkage peaks.**
(DOCX)

Figure S2 **Genome-wide autosomal multipoint linkage results for each biomarker (for those not already presented in main manuscript)**.
(DOCX)
