# Supplementary material for: Genome-Wide Linkage Analysis of Cardiovascular Disease Biomarkers in a Large, Multigenerational Family
Source: PLoS One. 2013 Aug 2;8(8):e71779. doi: 10.1371/journal.pone.0071779 (PMC3732259; doi:10.1371/journal.pone.0071779)
Supplement: Table S1 — Percent of samples measured as below lower limits of quantification for a given biomarker assay. (DOCX) [file pone.0071779.s001.docx]

**Table S1. Percent of samples measured as below lower limits of quantification for a given biomarker assay.**

| Biomarker | Percent of Sample Below Lower Limits of Quantification |
| --- | --- |
| Adiponectin | 1.1 |
| hsCRP | 0.0 |
| D-dimer | 0.3 |
| GCSF | 1.4 |
| GSP | 0.0 |
| IL1Rα | 1.4 |
| IL6 | 1.4 |
| IL8 | 1.4 |
| Leptin | 2.0 |
| MCP1 | 1.4 |
| MMP3 | 1.7 |
| Paraoxonase | 0.0 |
| RANTES | 1.4 |
| TNFα | 1.4 |
| TNFR1 | 1.4 |
| TNFR2 | 1.4 |
| TRAIL | 1.7 |
| VEGF | 1.4 |
