# Supplementary material for: Genome-Wide Linkage Analysis of Cardiovascular Disease Biomarkers in a Large, Multigenerational Family
Source: PLoS One. 2013 Aug 2;8(8):e71779. doi: 10.1371/journal.pone.0071779 (PMC3732259; doi:10.1371/journal.pone.0071779)
Supplement: Table S2 — Displayed are the maximum two-point LOD score at theta equal zero (MaxLOD) for each biomarker by chromosome, with the name of the probe (RS number), the centimorgan (cM) position of the probe, and the gene annotation for the SNP (or the closest gene for intergenic SNPs). (DOCX) [file pone.0071779.s002.docx]

**Table S2. Displayed are the maximum two-point LOD score at theta equal zero (MaxLOD) for each biomarker by chromosome, with the name of the probe (RS number), the centimorgan (cM) position of the probe, and the gene annotation for the SNP (or the closest gene for intergenic SNPs).**

| **Biomarker** | **Chr** | **MaxLOD** | **PROBE** | **Location**  **(cM)** | **Gene*** |
| --- | --- | --- | --- | --- | --- |
| **Adiponectin** | 1 | 0.74 | RS1334334 | 112.1 | Intergenic (LMO4, PKN2) |
|  | 2 | 0.86 | RS264963 | 117.4 | Intergenic (LOC644265, LOC100287010) |
|  | 3 | 1.05 | RS736357 | 52.9 | RBMS3 |
|  | 4 | 1.76 | RS1039180 | 161.2 | Intergenic (TLL1) |
|  | 5 | 0.60 | RS878781 | 69.0 | ARL15 |
|  | 6 | 1.17 | RS1349710 | 144.9 | Intergenic (NMBR) |
|  | 7 | 0.92 | RS4148738 | 99.4 | ABCB1 |
|  | 8 | 0.85 | RS1901386 | 57.8 | ADAM2 |
|  | 9 | 1.46 | RS717081 | 41.3 | Intergenic (SLC24A2, MLLT3) |
|  | 10 | 1.00 | RS1034178 | 123.5 | SORCS3 |
|  | 11 | 1.16 | RS1695 | 72.2 | GSTP1 |
|  | 12 | 1.06 | RS216293 | 16.4 | VWF |
|  | 13 | 0.54 | RS300320 | 58.1 | Intergenic (TDRD3) |
|  | 14 | 0.57 | RS1889387 | 29.3 | Intergenic (NUBPL) |
|  | 15 | 0.55 | RS1426932 | 44.0 | Intergenic (SPATA5L1) |
|  | 16 | 0.75 | RS11648785 | 130.8 | DBNDD1 |
|  | 17 | 0.55 | RS1230101 | 73.5 | ARHGAP27 |
|  | 18 | 1.07 | RS1015416 | 88.8 | SERPINB2 |
|  | 19 | 0.42 | RS11671074 | 70.9 | ZNF235 |
|  | 20 | 1.50 | RS1981431 | 69.4 | SDC4 |
|  | 21 | 1.15 | RS2825416 | 16.7 | Intergenic (SLC6A6P1) |
|  | 22 | 0.77 | RS1474834 | 68.5 | Intergenic (FAM19A5) |
| **hsCRP** | 1 | 1.15 | RS861212 | 40.6 | RAP1GAP |
|  | 2 | 1.95 | RS9973663 | 261.6 | GAL3ST2 |
|  | 3 | 1.43 | RS1545866 | 181.6 | Intergenic (NAALADL2) |
|  | 4 | 1.68 | RS6553000 | 197.4 | LOC2855441 |
|  | 5 | 0.73 | RS185312 | 108.0 | RHOBTB3 |
|  | 6 | 1.70 | RS773676 | 117.7 | LOC100652953 |
|  | 7 | 2.10 | RS1419607 | 128.2 | Intergenic (POT1, GRM8) |
|  | 8 | 1.27 | RS11992651 | 55.4 | Intergenic (UNC5D, KCNU1) |
|  | 9 | 1.96 | RS1491100 | 109.1 | Intergenic (TMEM38B, LOC644620) |
|  | 10 | 1.62 | RS10901726 | 158.2 | LOC728065 |
|  | 11 | 2.02 | RS728919 | 69.5 | NAA40 |
|  | 12 | 1.28 | RS990949 | 8.0 | Intergenic (LOC100128253, PRMT8) |
|  | 13 | 1.61 | RS1427167 | 52.0 | Intergenic (FNDC3A) |
|  | 14 | 1.28 | RS1012921 | 86.5 | Intergenic (GALC) |
|  | 15 | 1.03 | RS1567897 | 86.8 | Intergenic (MEX3B) |
|  | 16 | 1.37 | RS6993 | 76.2 | GOT2 |
|  | 17 | 1.33 | RS1057993 | 53.0 | CPD |
|  | 18 | 2.49 | RS7240966 | 69.9 | Intergenic (KIAA0427, SMAD7) |
|  | 19 | 1.80 | RS977708 | 55.8 | TDRD12 |
|  | 20 | 1.12 | RS4925370 | 108.3 | OSBPL2 |
|  | 21 | 0.89 | RS1155463 | 21.2 | Intergenic (NCAM2, NCRNA00158) |
|  | 22 | 1.35 | RS2075936 | 43.8 | CSF2RB |
| **DDimer** | 1 | 1.94 | RS488595 | 28.0 | FHAD1 |
|  | 2 | 1.28 | RS729386 | 123.6 | ACOXL |
|  | 3 | 1.70 | RS13975 | 131.8 | SLC12A8 |
|  | 4 | 1.31 | RS1115259 | 58.2 | FAM114A1 |
|  | 5 | 1.42 | RS895329 | 69.8 | Intergenic (SNX18, ESM1) |
|  | 6 | 1.14 | RS6597349 | 21.1 | LOC100506207 |
|  | 7 | 0.97 | RS258651 | 94.6 | CACNA2D1 |
|  | 8 | 1.65 | RS1872283 | 113.4 | Intergenic (ABRA, ANGPT1) |
|  | 9 | 2.18 | RS2780701 | 94.5 | SYK |
|  | 10 | 0.99 | RS1890876 | 33.4 | BEND7 |
|  | 11 | 0.88 | RS930983 | 127.5 | Intergenic (BSX) |
|  | 12 | 1.12 | RS717078 | 82.5 | Intergenic (DYRK2, IFNG) |
|  | 13 | 1.27 | RS1415708 | 90.9 | Intergenic (LOC121906, IPO5) |
|  | 14 | 1.23 | RS718212 | 66.5 | RDH12 |
|  | 15 | 1.30 | RS7168948 | 133.2 | Intergenic (TARSL2) |
|  | 16 | 1.19 | RS1482258 | 76.9 | Intergenic (GOT2) |
|  | 17 | 1.29 | RS7221818 | 15.0 | LOC339166 |
|  | 18 | 1.00 | RS7240966 | 69.9 | Intergenic (KIAA0427, SMAD7) |
|  | 19 | 0.93 | RS12151190 | 47.9 | RPSAP58 |
|  | 20 | 1.10 | RS466243 | 40.7 | MACROD2 |
|  | 21 | 0.71 | RS1808093 | 16.9 | Intergenic (LOC100505973) |
|  | 22 | 1.16 | RS5769127 | 63.0 | Intergenic (CERK) |
| **GCSF** | 1 | 1.37 | RS1416631 | 25.4 | Intergenic (PRDM2, KIAA1026) |
|  | 2 | 1.58 | RS728282 | 27.4 | NOL10 |
|  | 3 | 1.46 | RS1374679 | 86.3 | Intergenic (CADPS, LOC285401) |
|  | 4 | 1.59 | RS875579 | 12.9 | PPP2R2C |
|  | 5 | 0.51 | RS10903236 | 202.9 | Intergenic (ADAMTS2, RUFY1) |
|  | 6 | 0.85 | RS234915 | 6.1 | GMDS |
|  | 7 | 1.03 | RS1531381 | 180.1 | Intergenic (SHH, LOC389602) |
|  | 8 | 1.18 | RS935559 | 0.0 | C8orf42 |
|  | 9 | 1.33 | RS1327532 | 114.4 | Intergenic (TXNDC8, SVEP1) |
|  | 10 | 0.95 | RS2298033 | 37.7 | ITGA8 |
|  | 11 | 0.25 | RS622675 | 103.1 | ARHGAP42 |
|  | 12 | 0.96 | RS714480 | 28.2 | LOH12CR1 |
|  | 13 | 0.86 | RS1334958 | 0.0 | Intergenic (LOC100652856) |
|  | 14 | 0.68 | RS1468507 | 72.4 | LIN52 |
|  | 15 | 1.34 | RS2439378 | 68.3 | Intergenic (LCTL, SMAD6) |
|  | 16 | 0.96 | RS8045185 | 4.8 | CLCN7 |
|  | 17 | 0.88 | RS1063647 | 76.3 | PDK2 |
|  | 18 | 1.55 | RS869224 | 75.0 | DCC |
|  | 19 | 1.27 | RS10756 | 51.0 | URI1 |
|  | 20 | 1.37 | RS1058433 | 17.3 | GPCPD1 |
|  | 21 | 0.96 | RS2839377 | 78.7 | PRMT2 |
|  | 22 | 0.81 | RS2535694 | 4.1 | BCL2L13 |
| **GSP** | 1 | 0.84 | RS699778 | 141.0 | REG4 |
|  | 2 | 0.95 | RS1051783 | 116.4 | Intergenic (MFSD9) |
|  | 3 | 1.53 | RS1688378 | 111.4 | GABRR3 |
|  | 4 | 1.25 | RS1875863 | 98.3 | Intergenic (GPRIN3, SNCA) |
|  | 5 | 0.37 | RS29900 | 146.4 | KCTD16 |
|  | 6 | 0.96 | RS1321807 | 118.8 | ROS1 |
|  | 7 | 0.66 | RS2007475 | 63.4 | INHBA-AS1 |
|  | 8 | 0.58 | RS2203837 | 23.6 | KIAA1456 |
|  | 9 | 0.77 | RS1407392 | 113.2 | Intergenic (PALM2) |
|  | 10 | 0.64 | RS1547173 | 113.8 | Intergenic (PDE6C, C10orf4) |
|  | 11 | 0.62 | RS906895 | 9.8 | Upstream (CNGA4, CCKBR) |
|  | 12 | 0.81 | RS1508595 | 99.2 | Intergenic (KITLG) |
|  | 13 | 0.89 | RS625052 | 50.0 | LRRC63 |
|  | 14 | 0.46 | RS2891193 | 30.5 | Intergenic (ARHGAP5, AKAP6) |
|  | 15 | 0.66 | RS7168948 | 133.2 | Intergenic (TM2D3) |
|  | 16 | 0.50 | RS1018159 | 96.6 | WWOX |
|  | 17 | 0.28 | RS16523 | 67.8 | ARL5C |
|  | 18 | 1.05 | RS470473 | 115.1 | MBP |
|  | 19 | 0.56 | RS976282 | 82.1 | LOC100287477 |
|  | 20 | 0.50 | RS241605 | 11.9 | RNF24 |
|  | 21 | 0.69 | RS2825360 | 16.5 | Intergenic (LOC100505973) |
|  | 22 | 0.83 | RS1028503 | 67.6 | FAM19A5 |
| **IL1Ra** | 1 | 0.84 | RS4387226 | 126.2 | Intergenic (CDK4PS) |
|  | 2 | 0.64 | RS750365 | 216.0 | DIRC3 |
|  | 3 | 0.61 | RS1427434 | 94.1 | Intergenic (FRMD4B, MITF) |
|  | 4 | 0.56 | RS721412 | 113.8 | Intergenic (EGF, ELOVL6) |
|  | 5 | 0.60 | RS1544926 | 199.8 | COL23A1 |
|  | 6 | 0.66 | RS1986818 | 0.0 | Intergenic (FLJ43763) |
|  | 7 | 0.49 | RS6953748 | 188.9 | PTPRN2 |
|  | 8 | 0.88 | RS1125265 | 26.2 | SGCZ |
|  | 9 | 0.73 | RS2275241 | 134.1 | Intergenic (LMX1B) |
|  | 10 | 0.38 | RS3793670 | 177.4 | INPP5A |
|  | 11 | 0.81 | RS589149 | 81.6 | Intergenic (UVRAG, WNT11) |
|  | 12 | 1.39 | RS1492254 | 111.2 | ANKS1B |
|  | 13 | 0.60 | RS1411736 | 108.5 | Intergenic (LOC390424, FAM155A) |
|  | 14 | 0.80 | RS4982599 | 14.8 | Intergenic (TRAV38-2DV8) |
|  | 15 | 0.36 | RS2033610 | 28.9 | RYR3 |
|  | 16 | 0.61 | RS41103 | 113.2 | Intergenic (CDH13, HSBP1) |
|  | 17 | 0.67 | RS2941506 | 68.3 | PGAP3 |
|  | 18 | 0.84 | RS12959039 | 42.9 | Intergenic (CTAGE1, RBBP8) |
|  | 19 | 0.91 | RS440334 | 65.8 | Intergenic (CLC) |
|  | 20 | 0.50 | RS1570160 | 101.6 | LOC284757 |
|  | 21 | 0.40 | RS976531 | 63.5 | Intergenic (CRYAA) |
|  | 22 | 0.66 | RS713816 | 22.8 | MYO18B |
| **IL6** | 1 | 0.606 | RS1981193 | 121.82 | PALMD |
|  | 2 | 1.143 | RS935866 | 182.05 | Intergenic (H3F3AP4, CHRNA1) |
|  | 3 | 0.755 | RS1445111 | 49.7 | Intergenic (LRRC3B, NEK10) |
|  | 4 | 0.681 | RS920683 | 7.16 | ZBTB49 |
|  | 5 | 0.432 | RS1943049 | 167.321 | Intergenic (MAT2B, ODZ2) |
|  | 6 | 0.438 | RS1322669 | 68.02 | Intergenic (CDC5L) |
|  | 7 | 0.358 | RS1419607 | 128.2 | Intergenic (POT1, GRM8) |
|  | 8 | 0.336 | RS7827906 | 28.01 | Intergenic (MSR1, FGF20) |
|  | 9 | 0.786 | RS1331829 | 5.1 | Intergenic (SMARCA2) |
|  | 10 | 0.524 | RS1897620 | 76.17 | Intergenic (BICC1, PHYHIPL) |
|  | 11 | 0.645 | RS688030 | 64.95 | Intergenic (MS4A6A) |
|  | 12 | 0.564 | RS2730753 | 138.06 | Intergenic (SUDS3, KIAA1853) |
|  | 13 | 0.646 | RS898271 | 82.08 | Intergenic (LOC144776, MIRHG1) |
|  | 14 | 0.64 | RS1570342 | 15.26 | Intergenic (RBM23) |
|  | 15 | 0.389 | RS1562203 | 0 | Intergenic (TUBGCP5) |
|  | 16 | 0.195 | RS41103 | 113.21 | Intergenic (CDH13, HSBP1) |
|  | 17 | 0.458 | RS1848550 | 18.84 | Intergenic (TEKT1) |
|  | 18 | 0.486 | RS2045531 | 71.95 | MAPK4 |
|  | 19 | 0.342 | RS1019937 | 51.47 | Intergenic (C19orf2, ZNF536) |
|  | 20 | 0.91 | RS1535382 | 14.16 | RASSF2 |
|  | 21 | 0.204 | RS1014803 | 42.21 | Intergenic (LOC100506403) |
|  | 22 | 0.177 | RS713912 | 50.81 | EFCAB6 |
| **IL8** | 1 | 0.51 | RS7555879 | 17.8 | Intergenic (CASZ1, C1orf127) |
|  | 2 | 0.57 | RS7572888 | 81.7 | Intergenic (LOC644456, LOC730134) |
|  | 3 | 0.92 | RS338180 | 220.2 | DLG1 |
|  | 4 | 0.53 | RS951974 | 123.7 | TRPC3 |
|  | 5 | 0.58 | RS6891356 | 123.4 | Intergenic (RPL35AP15) |
|  | 6 | 0.69 | RS1180237 | 92.6 | ME1 |
|  | 7 | 0.55 | RS1468121 | 99.9 | Intergenic (ZNF804B) |
|  | 8 | 0.59 | RS2978328 | 4.5 | Intergenic (CSMD1) |
|  | 9 | 0.62 | RS12009 | 133.1 | HSPA5 |
|  | 10 | 0.77 | RS754575 | 173.2 | TCERG1L |
|  | 11 | 0.49 | RS1487718 | 62.9 | PRDM11 |
|  | 12 | 0.47 | RS1558776 | 15.6 | ANO2 |
|  | 13 | 0.47 | RS413756 | 119.0 | COL4A2 |
|  | 14 | 0.39 | RS11628338 | 8.8 | Intergenic (RNASE1) |
|  | 15 | 0.91 | RS2042613 | 22.6 | TRPM1 |
|  | 16 | 0.70 | RS30882 | 81.3 | Intergenic (CDH8, CDH11) |
|  | 17 | 0.57 | RS149245 | 10.0 | P2RX5 |
|  | 18 | 0.57 | RS9960471 | 89.0 | SERPINB8 |
|  | 19 | 0.44 | RS893179 | 110.8 | RPS5 |
|  | 20 | 0.31 | RS1981431 | 69.4 | SDC4 |
|  | 21 | 0.47 | RS1012959 | 43.7 | Intergenic (LOC728816, SIM2) |
|  | 22 | 0.60 | RS761793 | 67.4 | FAM19A5 |
| **Leptin** | 1 | 1.77 | RS10927583 | 27.2 | KAZN |
|  | 2 | 1.33 | RS5020134 | 3.0 | SNTG2 |
|  | 3 | 1.25 | RS669607 | 50.7 | Intergenic (EOMES, CMC1) |
|  | 4 | 1.04 | RS1317423 | 205.4 | Intergenic (TRIML1, FRG1) |
|  | 5 | 0.70 | RS1982301 | 204.3 | RASGEF1C |
|  | 6 | 0.81 | RS1771816 | 154.8 | MTHFD1L |
|  | 7 | 0.93 | RS441534 | 118.4 | NRCAM |
|  | 8 | 1.04 | RS12545053 | 75.8 | Intergenic (LOC100130155) |
|  | 9 | 1.22 | RS1143025 | 30.9 | ZDHHC21 |
|  | 10 | 1.77 | RS570967 | 167.7 | MGMT |
|  | 11 | 1.38 | RS1530891 | 71.9 | PC |
|  | 12 | 1.39 | RS897978 | 156.8 | TMEM132C |
|  | 13 | 1.07 | RS765244 | 4.2 | Intergenic (FGF9, LOC646201) |
|  | 14 | 0.61 | RS1547350 | 122.9 | KIF26A |
|  | 15 | 1.17 | RS288394 | 120.8 | Intergenic (LOC91948) |
|  | 16 | 0.88 | RS6993 | 76.2 | GOT2 |
|  | 17 | 1.12 | RS149245 | 10.0 | P2RX5 |
|  | 18 | 0.99 | RS650680 | 58.3 | CELF4 |
|  | 19 | 0.92 | RS1055099 | 69.4 | PHLDB3 |
|  | 20 | 0.85 | RS1342137 | 0.3 | DEFB126 |
|  | 21 | 0.81 | RS2837622 | 53.2 | DSCAM |
|  | 22 | 1.03 | RS713912 | 50.8 | EFCAB6 |
| **MCP1** | 1 | 2.14 | RS857819 | 153.2 | Intergenic (OR6K6, OR6N1) |
|  | 2 | 1.27 | RS714393 | 208.1 | ERBB4 |
|  | 3 | 1.05 | RS2076993 | 46.5 | Intergenic (LOC644990, LOC100130354) |
|  | 4 | 1.67 | RS1822842 | 140.1 | Intergenic (FREM3, GYPE) |
|  | 5 | 1.14 | RS878196 | 102.6 | Intergenic (CCNH) |
|  | 6 | 1.62 | RS1928533 | 69.7 | RUNX2 |
|  | 7 | 1.20 | RS7313 | 45.8 | CPVL |
|  | 8 | 0.98 | RS4628268 | 33.7 | CSGALNACT1 |
|  | 9 | 0.87 | RS855543 | 55.5 | Intergenic (ANKRD18B) |
|  | 10 | 1.03 | RS1928365 | 49.6 | GPR158 |
|  | 11 | 1.04 | RS160195 | 92.4 | GRM5 |
|  | 12 | 1.16 | RS1396208 | 45.1 | SOX5 |
|  | 13 | 1.16 | RS912278 | 51.9 | CYSLTR2 |
|  | 14 | 1.09 | RS718433 | 13.4 | Intergenic (TRAV6) |
|  | 15 | 1.79 | RS1463292 | 4.7 | Intergenic (NPAP1, SNRPN) |
|  | 16 | 0.99 | RS1019141 | 20.0 | RBFOX1 |
|  | 17 | 1.63 | RS1974692 | 87.6 | MSI2 |
|  | 18 | 1.05 | RS291781 | 42.6 | GREB1L |
|  | 19 | 1.70 | RS10410777 | 47.0 | Intergenic (LOC284441, LOC100288623) |
|  | 20 | 0.65 | RS756529 | 75.8 | KCNB1 |
|  | 21 | 0.92 | RS2254796 | 31.0 | LTN1 |
|  | 22 | 1.11 | RS10451 | 75.8 | SHANK3 |
| **MMP3** | 1 | 0.97 | RS491603 | 57.0 | Intergenic (EIF2C3, LOC100128093) |
|  | 2 | 1.04 | RS2001660 | 23.4 | IAH1 |
|  | 3 | 0.84 | RS326361 | 118.0 | Intergenic (CD47, UFT57) |
|  | 4 | 1.02 | RS3860640 | 184.5 | ODZ3 |
|  | 5 | 0.94 | RS1374080 | 34.1 | ANKH |
|  | 6 | 1.59 | RS11754333 | 46.4 | Intergenic (LOC389370, LOC100129616) |
|  | 7 | 1.07 | RS12217 | 125.8 | AASS |
|  | 8 | 1.19 | RS1481747 | 53.1 | NRG1 |
|  | 9 | 1.02 | RS954779 | 57.8 | Intergenic (RNF38, MELK) |
|  | 10 | 0.64 | RS4962480 | 155.0 | Intergenic (C10orf122, LOC100287412) |
|  | 11 | 1.44 | RS3345 | 146.7 | NTM |
|  | 12 | 1.54 | RS1563619 | 0.6 | Intergenic (NINJ2, LOC100132369) |
|  | 13 | 1.00 | RS4132778 | 7.1 | Intergenic (RS4132778) |
|  | 14 | 0.79 | RS1713419 | 4.3 | TEP1 |
|  | 15 | 0.77 | RS1871017 | 15.1 | Intergenic (GABRG3, OCA2) |
|  | 16 | 1.05 | RS1506842 | 92.1 | CNTNAP4 |
|  | 17 | 1.03 | RS759109 | 84.4 | ANKFN1 |
|  | 18 | 0.67 | RS625628 | 77.4 | Intergenic (TCF4, TXNL1) |
|  | 19 | 0.70 | RS759623 | 73.6 | CCDC61 |
|  | 20 | 0.77 | RS756529 | 75.8 | KCNB1 |
|  | 21 | 0.68 | RS990141 | 6.0 | Intergenic (SAMSN1) |
|  | 22 | 0.60 | RS2413411 | 42.2 | CACNG2 |
| **Paraoxonase** | 1 | 2.10 | RS491603 | 57.0 | Intergenic (EIF2C3, LOC100128093) |
|  | 2 | 1.88 | RS1504010 | 136.8 | CNTNAP5 |
|  | 3 | 1.19 | RS719300 | 139.7 | NPHP3-AS1 |
|  | 4 | 1.46 | RS1386117 | 75.5 | Intergenic (IGFBP7, LPHN3) |
|  | 5 | 1.30 | RS1047530 | 90.5 | 3’ UTR (F2RL2) |
|  | 6 | 1.25 | RS910563 | 93.4 | C6orf163 |
|  | 7 | 2.25 | RS234 | 115.4 | Intergenic (ATXN7L1, FLJ23834) |
|  | 8 | 1.88 | RS8685 | 54.2 | 3’ UTR (TTI2) |
|  | 9 | 1.13 | RS1329088 | 103.7 | LPPR1 |
|  | 10 | 1.14 | RS1892302 | 30.4 | CAMK1D |
|  | 11 | 1.41 | RS1508540 | 153.6 | B3GAT1 |
|  | 12 | 1.96 | RS872168 | 57.4 | Intergenic (PDZRN4) |
|  | 13 | 2.00 | RS726455 | 123.5 | Intergenic (LOC100287321, SOX1) |
|  | 14 | 0.72 | RS718433 | 13.4 | Intergenic (TRAV6) |
|  | 15 | 1.56 | RS2055893 | 8.7 | Intergenic (LOC100128714, LOC100289089) |
|  | 16 | 0.85 | RS2072042 | 3.5 | 3’ UTR (SOX8) |
|  | 17 | 1.42 | RS1046896 | 136.8 | 3’ UTR (FN3KRP) |
|  | 18 | 1.44 | RS1241983 | 22.6 | ARHGAP28 |
|  | 19 | 0.89 | RS1973371 | 47.9 | Intergenic (ZNF91) |
|  | 20 | 1.06 | RS2026162 | 106.4 | CDH4 |
|  | 21 | 0.84 | RS1155463 | 21.2 | Intergenic (NCAM2, NCRNA00158) |
|  | 22 | 0.89 | RS762058 | 37.9 | LARGE |
| **RANTES** | 1 | 1.04 | RS1886651 | 48.1 | Intergenic (PTPRU, LOC100288450) |
|  | 2 | 1.39 | RS1430197 | 79.6 | EFEMP1 |
|  | 3 | 0.82 | RS1030576 | 220.9 | Intergenic (BDH1, MIR922) |
|  | 4 | 1.58 | RS238510 | 107.3 | Intergenic (MTND5P5, SLC39A8) |
|  | 5 | 1.29 | RS1039322 | 161.8 | Intergenic (THG1L, CLINT1) |
|  | 6 | 1.26 | RS234915 | 6.1 | GMDS |
|  | 7 | 1.60 | RS7817 | 120.5 | IFRD1 |
|  | 8 | 1.09 | RS1464092 | 88.6 | Intergenic (NHF4G) |
|  | 9 | 0.66 | RS2031197 | 69.5 | RORB |
|  | 10 | 0.96 | RS11195400 | 128.6 | SHOC2 |
|  | 11 | 1.62 | RS601535 | 104.7 | DYNC2H1 |
|  | 12 | 1.17 | RS2058804 | 124.7 | KCTD10 |
|  | 13 | 1.02 | RS292476 | 6.9 | Intergenic (FGF9, LOC646201) |
|  | 14 | 0.76 | RS1959344 | 3.0 | Intergenic (OR11H6, OR11H4) |
|  | 15 | 1.11 | RS2739765 | 6.6 | SNORD115-15 |
|  | 16 | 0.98 | RS2239340 | 47.6 | PRKCB |
|  | 17 | 1.51 | RS1387545 | 136.4 | FOXK2 |
|  | 18 | 0.80 | RS1866338 | 118.8 | SALL3 |
|  | 19 | 1.20 | RS1017379 | 91.1 | Intergenic (ZNF613, ZNF350) |
|  | 20 | 0.95 | RS1570344 | 47.4 | Intergenic (SLC24A3) |
|  | 21 | 1.29 | RS2210267 | 53.1 | DSCAM |
|  | 22 | 1.40 | RS739240 | 71.4 | C22orf34 |
| **TNFa** | 1 | 0.71 | RS2744847 | 19.6 | Intergenic (UBIAD1, PTCHD2) |
|  | 2 | 0.73 | RS1569135 | 189.7 | Intergenic (IMPDH1P7, CALCRL) |
|  | 3 | 0.55 | RS1528198 | 85.1 | CADPS |
|  | 4 | 0.42 | RS1425566 | 131.2 | Intergenic (PCDH10) |
|  | 5 | 0.38 | RS4607330 | 113.0 | Intergenic (RAB9P1, LOC100287833) |
|  | 6 | 0.81 | RS1080085 | 171.4 | Intergenic (AGPAT4, PARK2) |
|  | 7 | 0.91 | RS2536077 | 164.8 | PRKAG2 |
|  | 8 | 0.42 | RS935559 | 0.0 | C8orf42 |
|  | 9 | 0.42 | RS1334071 | 123.7 | ASTN2 |
|  | 10 | 0.33 | RS713251 | 116.5 | TM9SF3 |
|  | 11 | 0.28 | RS10792360 | 64.0 | Intergenic (OR5L2, OR5D16) |
|  | 12 | 0.48 | RS2365567 | 18.4 | Intergenic (COPS7A) |
|  | 13 | 0.43 | RS1411736 | 108.5 | Intergenic (LOC390424, FAM155A) |
|  | 14 | 0.25 | RS715756 | 55.8 | PELI2 |
|  | 15 | 0.31 | RS1009913 | 42.7 | INO80 |
|  | 16 | 0.57 | RS237131 | 51.8 | Intergenic (HS3ST4, C16orf82) |
|  | 17 | 0.60 | RS16523 | 67.8 | ARL5C |
|  | 18 | 0.50 | RS872994 | 110.6 | Intergenic (C18orf62, LOC100289440) |
|  | 19 | 0.55 | RS479036 | 58.1 | LSM14A |
|  | 20 | 0.42 | RS2746097 | 57.2 | Intergenic (EPB41L1) |
|  | 21 | 0.65 | RS1378079 | 64.3 | HSF2BP |
|  | 22 | 0.33 | RS7286586 | 70.4 | Intergenic (LOC100287247, FLJ44385) |
| **TNFR1** | 1 | 0.32 | RS1938584 | 97.4 | LRRC7 |
|  | 2 | 0.33 | RS934748 | 125.3 | Intergenic (POLR1B, SLC20A1) |
|  | 3 | 0.37 | RS1515628 | 165.4 | SCHIP1 |
|  | 4 | 0.42 | RS729918 | 35.7 | SLIT2 |
|  | 5 | 0.29 | RS720846 | 17.9 | NSUN2 |
|  | 6 | 0.34 | RS1486039 | 88.8 | Intergenic (IMPG1, HTR1B) |
|  | 7 | 0.30 | RS11178 | 110.4 | SERPINE1 |
|  | 8 | 0.26 | RS11992651 | 55.4 | Intergenic (UNC5D, KCNU1) |
|  | 9 | 0.21 | RS88644 | 85.5 | Intergenic (ZCCHC6, GAS1) |
|  | 10 | 0.43 | RS754575 | 173.2 | TCERG1L |
|  | 11 | 0.24 | RS1027323 | 139.5 | Intergenic (RICS, BARX2) |
|  | 12 | 0.31 | RS1506918 | 74.3 | Intergenic (LOC100289417, FAM19A2) |
|  | 13 | 0.52 | RS1008812 | 8.8 | SACS |
|  | 14 | 0.39 | RS17461158 | 20.7 | Intergenic (STXBP6, NOVA1) |
|  | 15 | 0.34 | RS2042613 | 22.6 | TRPM1 |
|  | 16 | 0.25 | RS1874014 | 117.3 | CRISPLD2 |
|  | 17 | 0.26 | RS116719 | 116.5 | MFSD11 |
|  | 18 | 0.18 | RS593418 | 94.7 | Intergenic (DSEL, TMX3) |
|  | 19 | 0.27 | RS870379 | 60.3 | HPN |
|  | 20 | 0.24 | RS6054770 | 23.3 | Intergenic (BMP2, HAO1) |
|  | 21 | 0.10 | RS2837956 | 55.3 | Intergenic (DSCAM, C21orf130) |
|  | 22 | 0.20 | RS756658 | 7.9 | UFD1L |
| **TNFR2** | 1 | 0.76 | RS3845516 | 270.8 | SMYD3 |
|  | 2 | 0.55 | RS4669630 | 27.8 | PDIA6 |
|  | 3 | 0.44 | RS1472621 | 128.3 | Intergenic (CASR, CSTA) |
|  | 4 | 0.54 | RS1557816 | 19.0 | AFAP1 |
|  | 5 | 0.38 | RS726847 | 138.5 | Intergenic (TRPC7, SPOCK1) |
|  | 6 | 0.50 | RS736794 | 61.3 | UNC5CL |
|  | 7 | 0.43 | RS1882600 | 13.6 | Intergenic (C1GALT1, COL28A1) |
|  | 8 | 0.56 | RS13267098 | 82.7 | Intergenic (XKR9) |
|  | 9 | 0.26 | RS877954 | 151.7 | RXRA |
|  | 10 | 0.55 | RS4065 | 94.0 | 3’ UTR (PLAU) |
|  | 11 | 0.43 | RS920443 | 57.2 | LRRC4C |
|  | 12 | 0.62 | RS933552 | 22.4 | SLC2A3 |
|  | 13 | 0.36 | RS1164591 | 89.0 | HS6ST3 |
|  | 14 | 0.28 | RS1740696 | 96.4 | ITPK1 |
|  | 15 | 0.52 | RS12442894 | 88.6 | HOMER2 |
|  | 16 | 0.54 | RS1566467 | 58.3 | Intergenic (SIAH1, N4BP1) |
|  | 17 | 0.62 | RS1872087 | 109.0 | SDK2 |
|  | 18 | 0.48 | RS872994 | 110.6 | Intergenic (C18orf62, LOC100289440) |
|  | 19 | 0.42 | RS1017379 | 91.1 | Intergenic (ZNF613, ZNF350) |
|  | 20 | 0.27 | RS736264 | 57.1 | Intergenic (CHMP4B) |
|  | 21 | 0.36 | RS2838970 | 73.3 | Intergenic (SLC19A1) |
|  | 22 | 0.32 | RS2252257 | 5.9 | USP18 |
| **TRAIL** | 1 | 0.67 | RS3818157 | 21.3 | PLOD1 |
|  | 2 | 0.86 | RS13032599 | 163.6 | GALNT13 |
|  | 3 | 0.75 | RS1401866 | 168.5 | Intergenic (BCHE, LOC131055) |
|  | 4 | 0.89 | RS1921847 | 92.3 | Intergenic (AGPAT9, LOC152845) |
|  | 5 | 0.55 | RS1394641 | 22.0 | Intergenic (MIR4458, MIR4636) |
|  | 6 | 0.74 | RS2032985 | 14.1 | FARS2 |
|  | 7 | 0.81 | RS1558557 | 16.3 | Intergenic (ICA1, NXPH1) |
|  | 8 | 0.75 | RS6468383 | 55.4 | KCNU1 |
|  | 9 | 0.49 | RS1556031 | 30.5 | Intergenic (NFIB, ZDHHC21) |
|  | 10 | 0.46 | RS1969825 | 21.0 | SFMBT2 |
|  | 11 | 0.42 | RS575030 | 124.5 | Intergenic (TECTA, SC5DL) |
|  | 12 | 0.92 | RS617022 | 14.9 | Intergenic (KCNA5) |
|  | 13 | 0.44 | RS53248 | 108.7 | Intergenic (FAM155A) |
|  | 14 | 0.68 | RS1022590 | 106.4 | Intergenic (VRK1, C14orf64) |
|  | 15 | 0.67 | RS1648312 | 43.9 | DUOX1 |
|  | 16 | 0.65 | RS189378 | 57.1 | Intergenic |
|  | 17 | 0.63 | RS1387545 | 136.4 | FOXK2 |
|  | 18 | 0.83 | RS1662910 | 58.0 | CELF4 |
|  | 19 | 0.89 | RS1019937 | 51.5 | Intergenic (C19orf2, ZNF536) |
|  | 20 | 0.57 | RS1570160 | 101.6 | Intergenic (LOC284757) |
|  | 21 | 0.70 | RS926130 | 6.4 | Intergenic (LOC388813, NRIP1) |
|  | 22 | 0.50 | RS1557622 | 3.3 | Intergenic (CECR1, CECR2) |
| **VEGF** | 1 | 1.80 | RS1912915 | 189.2 | Intergenic (PLA2G4A, FAM5C) |
|  | 2 | 1.73 | RS7560042 | 163.2 | Intergenic (RPRM, GALNT13) |
|  | 3 | 1.10 | RS1437049 | 109.2 |  |
|  | 4 | 2.56 | RS790142 | 61.0 | Intergenic (RBM47, LOC100289402) |
|  | 5 | 1.88 | RS756757 | 22.0 | Intergenic (LOC729506, LOC100128382) |
|  | 6 | 1.85 | RS2025157 | 147.9 | Intergenic (GRM1, RAB32) |
|  | 7 | 1.47 | RS757723 | 161.3 | Intergenic (KRBA1) |
|  | 8 | 1.39 | RS569950 | 35.9 | Intergenic (LZTS1, GFRA2) |
|  | 9 | 1.35 | RS1332184 | 42.2 | Intergenic (IFNE, LOC554202) |
|  | 10 | 1.16 | RS4935215 | 70.2 | PRKG1 |
|  | 11 | 1.76 | RS592955 | 107.8 | ATM |
|  | 12 | 1.15 | RS1344945 | 78.8 | MSRB3 |
|  | 13 | 1.72 | RS625052 | 50.0 | LRRC63 |
|  | 14 | 1.23 | RS7160965 | 28.0 | AP4S1 |
|  | 15 | 0.95 | RS1009913 | 42.7 | INO80 |
|  | 16 | 1.05 | RS741720 | 43.4 | Intergenic (ZP2, ANKS4B) |
|  | 17 | 1.67 | RS917310 | 41.4 | Intergenic (HS3ST3B1) |
|  | 18 | 1.59 | RS593418 | 94.7 | Intergenic (DSEL, TMX3) |
|  | 19 | 1.45 | RS1017379 | 91.1 | Intergenic (ZNF613, ZNF350) |
|  | 20 | 1.68 | RS434609 | 0.5 | DEFB132 |
|  | 21 | 0.92 | RS767055 | 22.0 | Intergenic (NCAM2, NCRNA00158) |
|  | 22 | 0.61 | RS2024566 | 49.3 | Intergenic (RANGAP1, ZC3H7B) |
