# Supplementary figures and images for: Genome-Wide Linkage Analysis of Cardiovascular Disease Biomarkers in a Large, Multigenerational Family
Source: PLoS One. 2013 Aug 2;8(8):e71779. doi: 10.1371/journal.pone.0071779 (PMC3732259; doi:10.1371/journal.pone.0071779)

Figure S1a


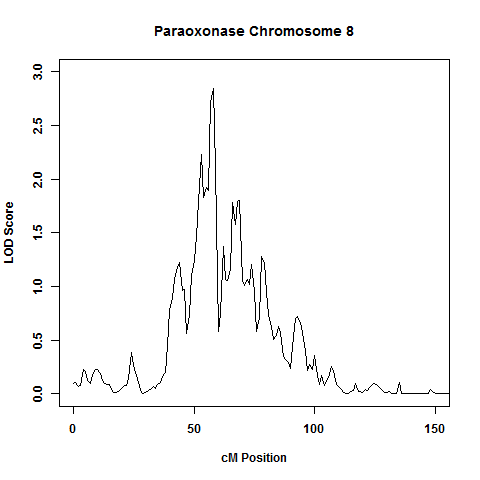


Figure S1b


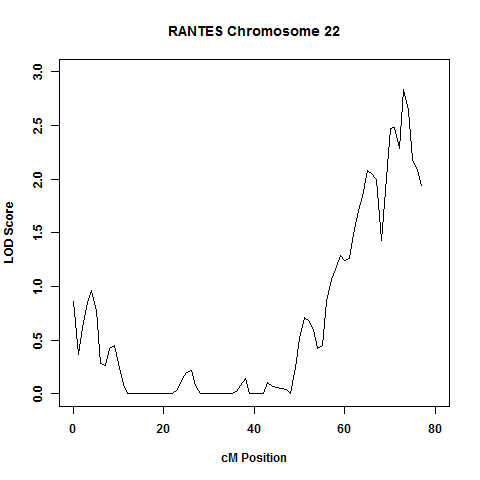


Figure S1c


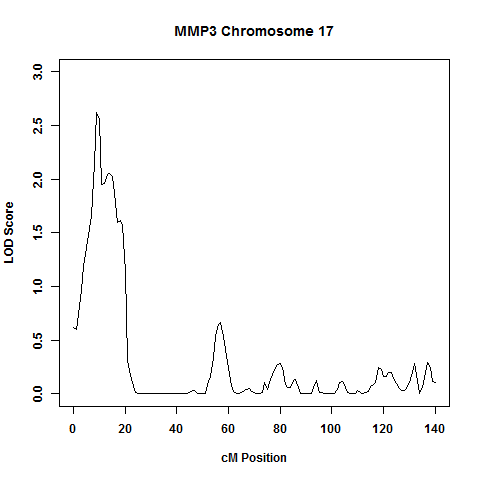


Figure S1d


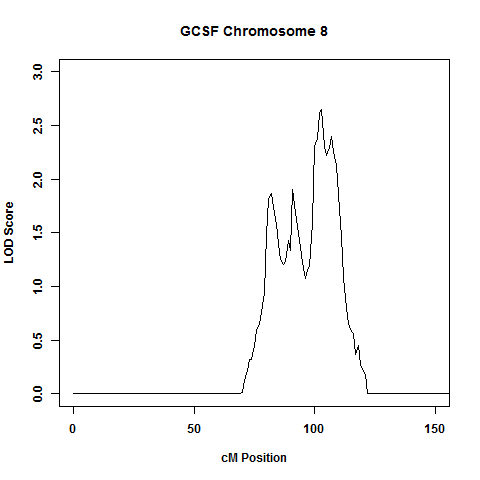

Supplement: Figure S1 — Chromosome linkage plots for the most significant multipoint linkage peaks. (DOCX) [file pone.0071779.s003.docx]
