# Supplementary material for: Genome-Wide Linkage Analysis of Cardiovascular Disease Biomarkers in a Large, Multigenerational Family
Source: PLoS One. 2013 Aug 2;8(8):e71779. doi: 10.1371/journal.pone.0071779 (PMC3732259; doi:10.1371/journal.pone.0071779)

**Figure S2a Adiponectin**


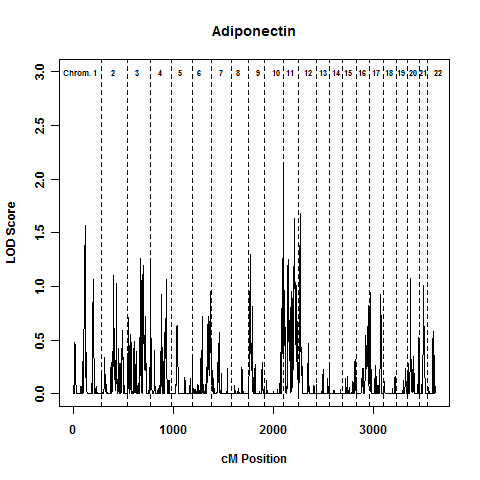


**Figure S2b hsCRP**


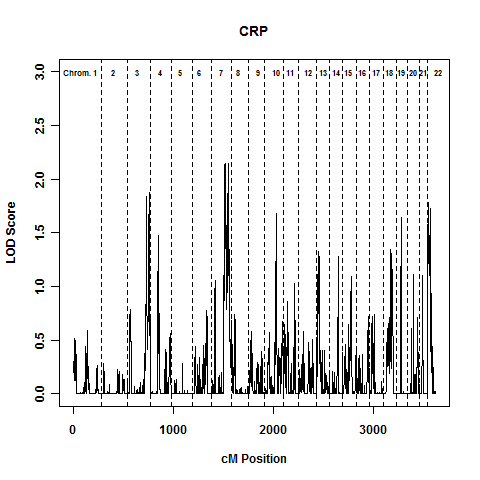


**Figure S2c D-dimer**


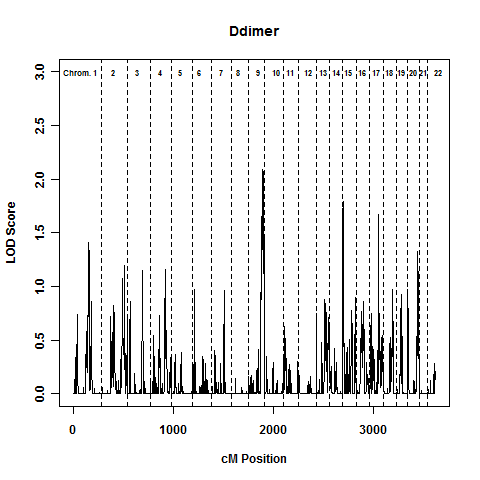


**Figure S2d GSP**


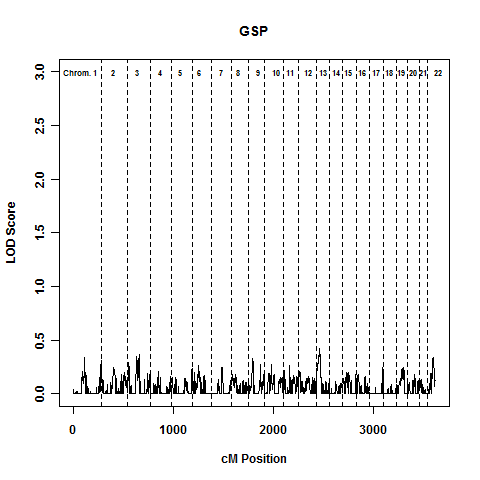


**Figure S2e IL-6**


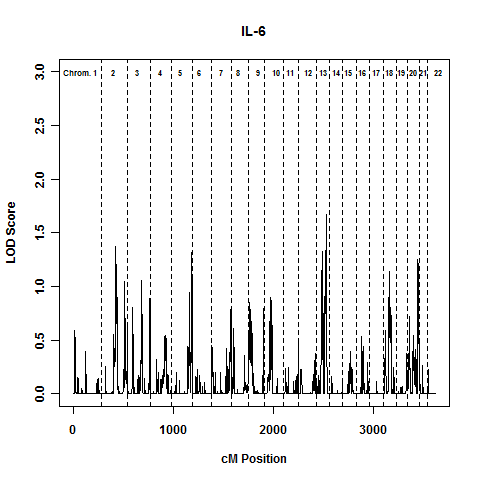


**Figure S2f IL-8**


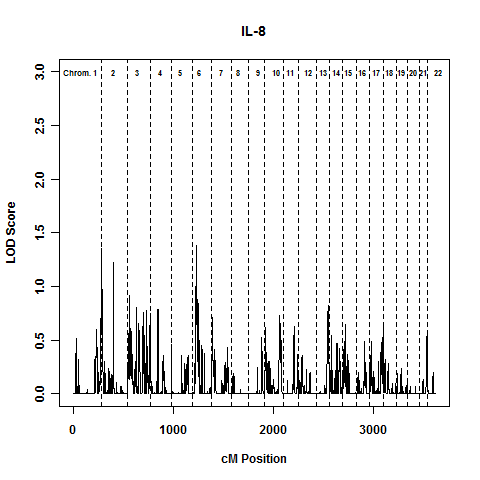


**Figure S2g IL1Ra**


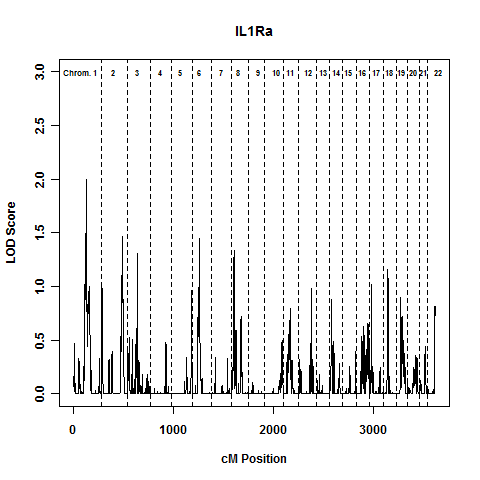


**Figure S2h Leptin**


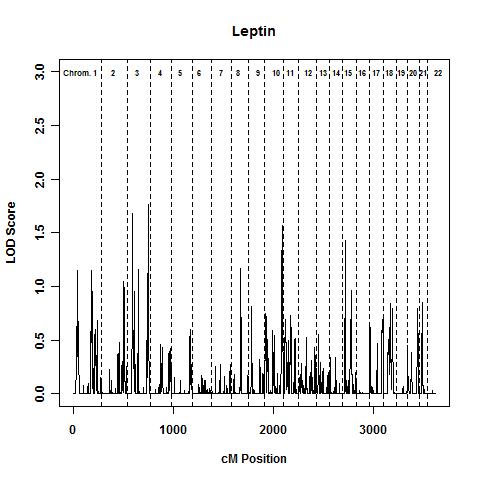


**Figure S2i MCP1**


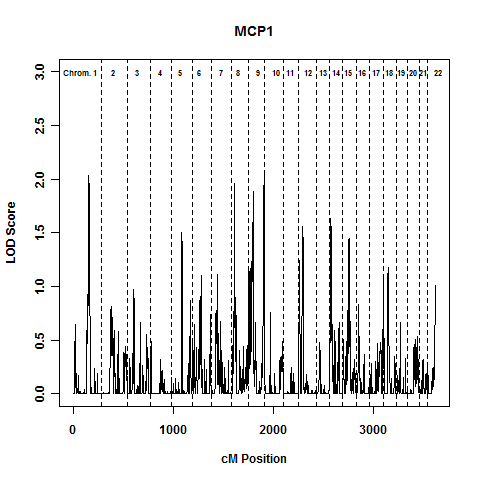


**Figure S2j TNFα**


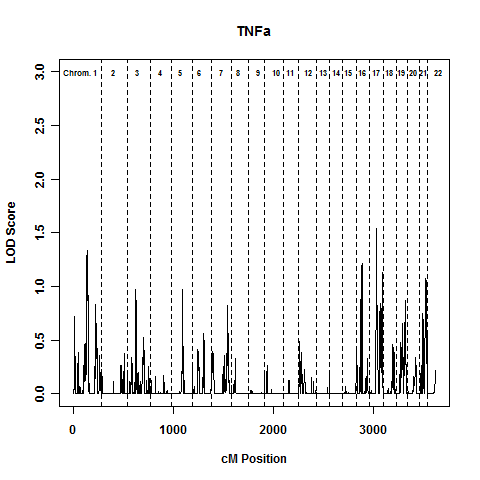


**Figure S2k TNFR2**


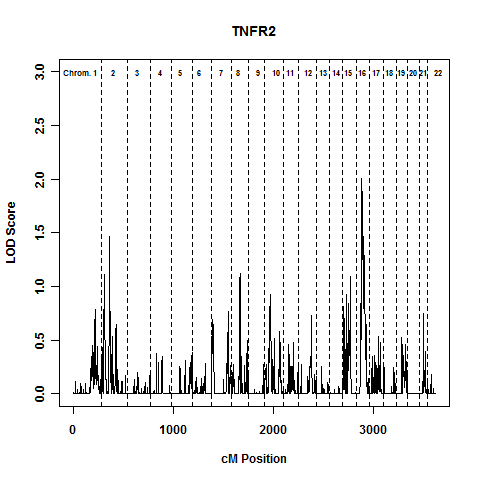


**Figure S2l TNFR1**


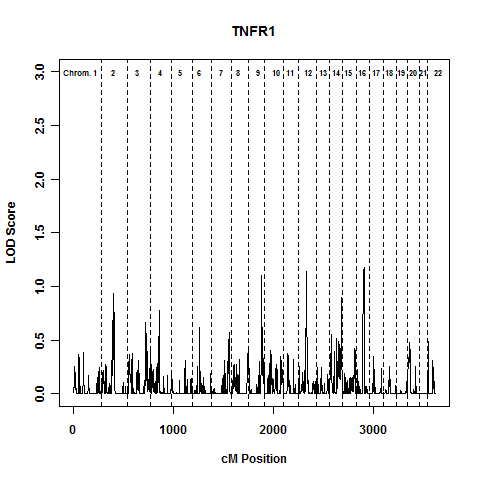


**Figure S2m TRAIL**


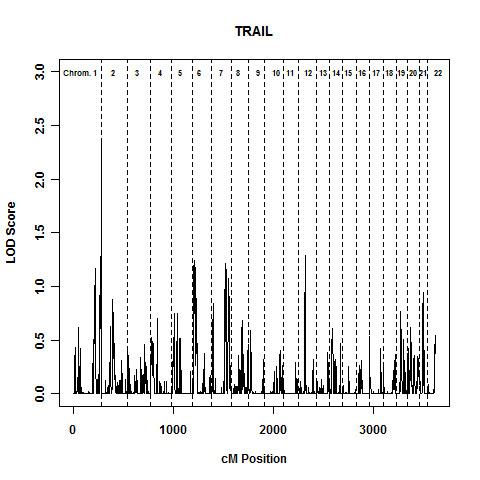


**Figure S2n VEGF**


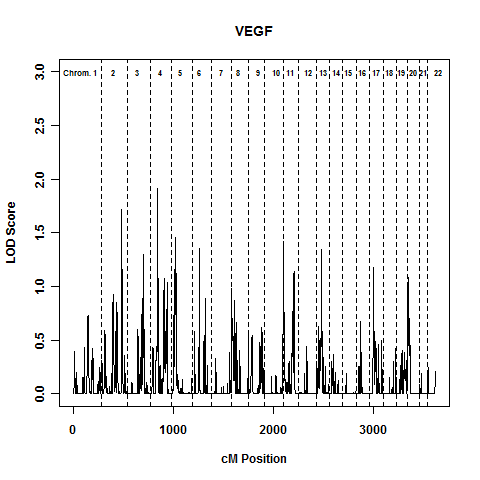

Supplement: Figure S2 — Genome-wide autosomal multipoint linkage results for each biomarker (for those not already presented in main manuscript). (DOCX) [file pone.0071779.s004.docx]
